# Trehalose enhances neuronal differentiation with VEGF secretion in human iPSC-derived neural stem/progenitor cells

**DOI:** 10.1016/j.reth.2025.06.012

**Published:** 2025-06-26

**Authors:** Sean K. Roose, Yoichi Mizukami, Jun Muto, Hideyuki Okano, Masaya Nakamura, Narihito Nagoshi

**Affiliations:** a Department of Orthopaedic Surgery, Keio University School of Medicine, 35 Shinanomachi, Shinjuku-Ku, Tokyo 160-8582, Japan; b Institute of Gene Research, Yamaguchi University Science Research Center, 1-1-1 MinamiKogushi, Ube-Shi, Yamaguchi 755-8505, Japan; c Department of Dermatology, Ehime University Graduate School of Medicine, 454 Shitsukawa, Touon-Shi, Ehime 791-0295, Japan; d Keio University Regenerative Medicine Research Center, 3-25-10 Tonomachi, Kawasaki-Ku, Kawasaki, Kanagawa 210-0821 Japan

**Keywords:** Induced pluripotent stem cells, Neural stem/progenitor cells, Trehalose, Neuronal differentiation, Vascular endothelial growth factor, SOX2

## Abstract

**Introduction:**

Cell transplantation therapy has emerged as a promising approach in regenerative medicine for treating neurological diseases. Neural stem/progenitor cell (NS/PC) transplantation has demonstrated therapeutic efficacy; however, its potential remains limited by suboptimal differentiation and insufficient secretion of pro-healing growth factors. Trehalose, a glucose disaccharide, has been shown to exert neuroprotective effects by inducing autophagy and stabilizing cellular structures. Recent studies suggest that trehalose can modulate growth factor secretion through the CDKN1A/p21 pathway. However, its impact on human induced pluripotent stem cell-derived NS/PCs (hiPSC-NS/PCs) remains unclear. This study investigates the effect of trehalose on neuronal differentiation, cell viability, and growth factor expression in hiPSC-NS/PCs to explore its potential in enhancing transplantation therapy.

**Methods:**

hiPSC-NS/PCs were cultured as neurospheres and treated with trehalose (10 mg/ml or 40 mg/ml) for 7 days. Cell viability was assessed using CellTiter Glo® assay. Gene expression analysis was conducted via qRT-PCR and RNA-seq, particularly focusing on *CDKN1A*, *VEGFA*, *FGF2*, and *BDNF*. Protein expression of SOX2 was analyzed via western blotting. Neurite outgrowth was evaluated using MAP2 immunostaining following differentiation. Statistical significance was set at p < 0.05.

**Results:**

Treatment with 10 mg/ml trehalose upregulated *CDKN1A* expression and promoted neuronal differentiation, as evidenced by reduced SOX2 expression and enhanced neurite outgrowth. RNA-seq analysis revealed the activation of growth factor-related pathways, including *VEGFA* upregulation, which persisted even after trehalose withdrawal (p = 0.016). However, high concentration (40 mg/ml) significantly reduced cell viability (p = 0.032), suggesting dose-dependent cytotoxicity.

**Conclusion:**

Trehalose enhances neuronal differentiation and *VEGFA* secretion in hiPSC-NS/PCs, potentially augmenting the efficacy of transplantation therapy. These findings suggest that trehalose may serve as a valuable adjunct for neural regeneration, though optimal dosing must be determined to balance differentiation enhancement and cell viability. Further *in vivo* studies are warranted to validate its clinical applicability.

## Highlights

• Trehalose promotes neuronal differentiation in human induced pluripotent stem cell-derived neural stem/progenitor cells.
• Trehalose upregulates the long-term expression of VEGFA, an angiogenic and neurogenic growth factor.
• These findings suggest that trehalose could improve the efficacy of cell transplantation therapy for neurological diseases.

## 1 Introduction

Recent developments in regenerative medicine have highlighted the importance of cell transplantation therapy. Numerous studies have reported the efficacy of neural stem/progenitor cell (NS/PC) transplantation against neurological diseases and trauma [[1], [2], [3], [4]]. However, functional regeneration achieved by cell transplantation alone is still limited, and further research is needed to enhance the therapeutic benefits of NS/PCs, such as its ability to differentiate and secrete pro-healing growth factors.

Trehalose is a common glucose disaccharide found endogenously in many organisms, including invertebrates, fungi, bacteria, and plants [5]. It is a non-reducing, stable substance, and its natural ability to stabilize proteins, enzymes, and tissues has been extensively studied in the context of neuroprotection. Many studies have reported that trehalose acts as an autophagy inducer, which promotes the clearance of pathogenic proteins in neurons of chronic neurological diseases such as Huntington disease, Parkinson disease, and Alzheimer's disease [[6], [7], [8]]. Furthermore, trehalose can protect the cells from neurological damage by stabilizing the bilayer structure of the cell membrane, mitigating oxidative stress, and inhibiting the release of proinflammatory cytokines [9,10]. Although the cell type was different, a recent study using fibroblasts found that trehalose at high concentration accelerates wound repair through secretion of multiple growth factors [11]. Moreover, the use of high concentration trehalose in *Zygosaccharomyces rouxii* yeast cell culture enhanced the cell's heat shock resistance without affecting glucose metabolism [12]. However, the effect of trehalose on human induced pluripotent stem cell-derived NS/PC (hiPSC-NS/PC) remains to be elucidated.

In this study, we evaluated the protocols of applying trehalose in neurosphere culture and investigated the effects of trehalose on hiPSC-NS/PCs with the aim of improving its therapeutic efficacy in cell transplantation therapy.

## 2 Methods

### 2.1 Establishment of hiPSC-NS/PCs

The hiPSC-NS/PCs used in this study were produced by a Good Manufacturing Practice (GMP)-grade cell processing facility at Osaka National Hospital, Japan from hiPSC line YZWJs513 [13], and were frozen for the purpose of conservation at 7th passage. The hiPSC line YZWJs513 was established at Center for iPS Cell Research and Application (CiRA) in Kyoto University.

### 2.2 Cell culture

Throughout this study, the hiPSC-NS/PCs were cultured using the neurosphere culture technique [14], in DMEM/Ham's F12 (FUJIFILM Wako Pure Chemical Corporation, Japan) supplemented with Heparin (Yoshindo, Japan), B-27 (Thermo Fisher Scientific, USA), bFGF (R&D Systems, USA), EGF (R&D Systems), and LIF (Creative BioMart, USA). We refer to this culture medium as NS/PC expansion medium.

All cells used in this study were thawed at a density of 1.8 × 10^5^ cells/ml in a T75 flask (Corning, USA), and cultured without trehalose in NS/PC expansion medium for 7 days at 37 °C in 5 % CO_2_ and 95 % air. The cells were then passaged at a density of 1.25 × 10^5^ cells/ml in 24-well plates (Corning), 6-well plates (Corning), or T25 flasks (Corning), and cultured using NS/PC expansion medium with or without trehalose (10 and 40 mg/ml or 10 mg/ml; Hayashibara, Okayama, Japan) for 7 days at 37 °C in 5 % CO_2_ and 95 % air before performing further experiments.

### 2.3 Viability assays

The number of alive cells in each well was counted using CellTiter Glo® 3D Cell Viability Assay (Promega, USA) and Enspire® Multimode Plate Reader (PerkinElmer, USA), following the manufacturer's instructions.

### 2.4 Phase contrast microscopy

Images of the cells were taken through phase contrast microscopy using IX73 Inverted Microscope (Olympas, Japan).

### 2.5 Gene expression analysis by qRT-PCR

Total RNA from each sample was extracted using the RNeasy Mini Kit (Qiagen, Germany), and cDNA was synthesized using the ReverTra Ace® qPCR RT Master Mix (TOYOBO, Japan) following the manufacturer's instructions. TaqMan® Gene Expression Assays were used to perform the qRT-PCR for measuring mRNA abundance. The primers used are listed in Table 1. ACTB mRNA was used as an internal control, and target gene mRNA expression was calculated by $2^{-\Delta \Delta Ct}$ method.

**Table 1:** TaqMan primers and probe assay ID used in qRT-PCR.

Gene	Assay ID
*CDKN1A*	Hs00355782_m1
*VEGFA*	Hs00900055_m1
*FGF2*	Hs00266645_m1
*BDNF*	Hs00542425_s1
*ACTB*	Hs01060665_g1

### 2.6 RNA-seq

Total RNA from each sample was isolated using the RNeasy Mini Kit (Qiagen), and mRNA was purified with oligo dT beads using NEBNext Poly(A) mRNA Magnetic Isolation Module (New England Biolabs, NEB, USA). RNA-seq library was prepared with NEBNext Ultra II Directional RNA Library Prep Kit for Illumina (NEB). Briefly, the isolated poly (A) RNA was fragmented using NEBNext Random Primers in NEBNext First Strand Synthesis Reaction Buffer, and reverse transcription was performed with NEBNext Strand Synthesis Enzyme Mix. The synthesized cDNA was ligated with NEBNext Adaptor (NEB), purified with AMPureXP (Beckman coulter, USA), and index sequences were inserted with PCR amplification using NEBNext Oligos for Illumina (NEB). The libraries were sequenced on Illumina NovaSeq 6000 (Illumina, USA) using NovaSeq 6000 S1 Reagent Kit V1.5 (Illumina), and data analysis was performed using CLC Genomics Workbench 23.0.2 (Qiagen). Pathway analysis was performed using IPA (Qiagen).

### 2.7 Western blotting analysis

Cells were lysed in RIPA buffer (Thermo Fisher Scientific) supplemented with protease inhibitor (1:100, Thermo Fisher Scientific) and phosphatase inhibitor (1:100, nacalai tesque). Proteins and Chameleon® Duo Pre-stained Protein Ladder (LICORbio, USA) were loaded and electrophoretically separated on 4–20 % gradient polyacrylamide gel. The proteins were then transferred onto a nitrocellulose membrane using the Trans-Blot Turbo Transfer System (Bio-Rad, USA), and was washed with TBS for 2 min. Normalization was done using Revert™ 700 Total Protein Stain (LICORbio) following the manufacturer's instructions. The membrane was blocked with Intercept Blocking Buffer TBS (LICORbio) for 1 h at room temperature, and incubated overnight at 4 °C with primary antibody against Sox2 (mouse IgG2a, 1:500, MAB2018, R&D Systems). After the membrane was washed with TBST 4 times, it was incubated with IRDye® 680RD Donkey anti-Mouse IgG Secondary Antibody (LICORbio) for 1 h at room temperature. The membrane was then washed with TBST for 4 times, washed with TBS once, and the bands were visualized using Odyssey CLx Imager (LICORbio).

### 2.8 In vitro differentiation

The neurospheres cultured with and without trehalose were separated into single cells using TrypLE™ select (Thermo Fisher Scientific). The cells were seeded in KBM Neural Stem Cell (Kohjin Bio, Japan) containing Penicillin-Streptomycin Mixed solution (0.5 %; Nacalai Tesque, Japan) and B27 (2 %; Thermo Fisher Scientific) at a density of 3.0 × 10^5^ cells/3.83 cm^2^ onto a poly-l-Lysine (Sigma Aldrich, USA) and Matrigel (Growth Factor Reduced, Corning)-coated 12 well plate (Corning). Y-27632 (0.1 %; nacalai tesque) was added only on the day of the seeding. The medium was exchanged every 3 days, and the differentiation was continued for 14 days at 37 °C in 5 % CO_2_ and 95 % air before total RNA was extracted.

A similar experiment was performed, except the cells were seeded at a density of 1.0 × 10^4^ cells/1.8 cm^2^ onto a poly-l-Lysine and Matrigel-coated cover glass using flexiPERM® (Sarstedt, Germany), and the differentiation was continued for 3 days before neurite outgrowth assay.

### 2.9 Neurite outgrowth assay

The differentiated cells on slide glass were washed with PBS once, fixed with 4 % PFA at 4 °C for 72 h, washed with PBS 3 times, blocked for 1 h at room temperature with a blocking solution (5 % skim milk, 1 % BSA, 0.1 % Triton X-100, PBS), washed with PBS once, and incubated at 4 °C over night with primary antibody solution containing MAP2 (Rabbit, 1:3000, AB5622, Millipore, USA). The cells were washed with PBS 3 times, and incubated for 1 h at room temperature with an Alexa Fluor-conjugated secondary antibody (1:800, Thermo Fisher) and Hoechst 33258 (1:1000, Sigma Aldrich). The cells were then washed with PBS 3 times and fixed with slide glass. Images of 5 regions per each sample were randomly acquired at room temperature using a confocal laser-scanning microscope (LSM780; Carl Zeiss, Germany). The length of the longest neurite per every MAP2 positive cell in each image was measured manually using Image J software (NIH Image J Software) [15]. The mean length of the 5 images for each sample was used for statistical analysis.

### 2.10 Statistical methods

Statistical analysis was performed with SPSS statistics (version 29.0.0.0; Japan IBM, Japan). Results are expressed as mean ± SEM. Differences were considered significant at *p* < 0.05. R Statistical Software (version 4.4.2; R Core Team 2024) [16] and ggplot2 R package (version 3.5.1; H. Wickham) [17] was used to create all figures except for RNA-seq analysis data.

## 3 Results

### 3.1 Trehalose reduces cell viability and upregulates CDKN1A expression

**Fig. 1:**
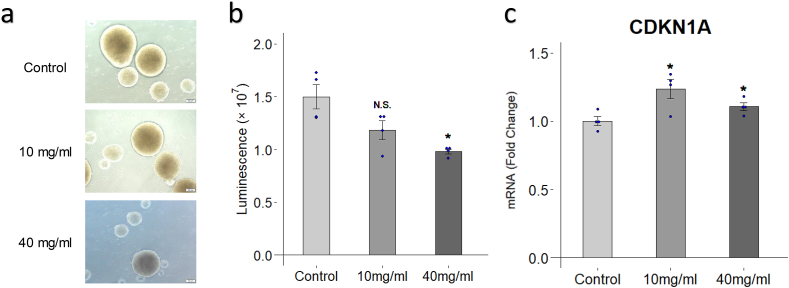
**Effect of trehalose on cell viability and CDKN1A expression. a** Representative image of neurospheres cultured with or without trehalose (10 and 40 mg/ml) for 7 days. Phase contrast micrographs, scale bar = 100 μm. **b** Cell viability was assessed by viability assay after hiPSC-NS/PCs were cultured with or without trehalose (10 and 40 mg/ml) for 7 days. Data were shown as luminescence which is proportional to the number of viable cells. n = 4 each. **c** CDKN1A expression was assessed by qRT-PCR after hiPSC-NS/PCs were cultured with or without trehalose (10 and 40 mg/ml) for 7 days. Data were shown as relative expressions to the control. n = 3 each. ∗p < 0.05 versus the control group according to Dunn-Bonferroni test in **a**, and one-way ANOVA followed by Bonferroni test in **c**. Data are presented as the mean ± SEM.

First, we performed viability assays on hiPSC-NS/PCs cultured with or without trehalose (0, 10, and 40 mg/ml) for 7 days (Fig. 1a and b). The results revealed that trehalose at 40 mg/ml caused a significant decrease in the number of viable cells (1.50 × 10^7^ vs. 9.80 × 10^6^, p = 0.032). Although trehalose at 10 mg/ml tended to have lower cell viability compared to control, there was no statistical difference (1.50 × 10^7^ vs. 1.18 × 10^7^, p = 0.607).

Next, we used quantitative reverse transcription polymerase chain reaction (qRT-PCR) to observe the mRNA expressions of *CDKN1A* on hiPSC-NS/PCs treated by trehalose (10 and 40 mg/ml) for 7 days (Fig. 1c). *CDKN1A* is the gene coding the cyclin-dependent kinase inhibitor 1A (p21), a protein involved in cell cycle regulation that is known to induce cell cycle arrest [18]. The cells treated by trehalose showed significant increase of *CDKN1A* expression in both concentrations compared to the control without trehalose (10 mg/ml: 1.26 vs. 1.00, p = 0.002; 40 mg/ml: 1.18 vs. 1.00, p = 0.014).

### 3.2 Trehalose promotes neuronal differentiation and cell cycle arrest

**Fig. 2:**
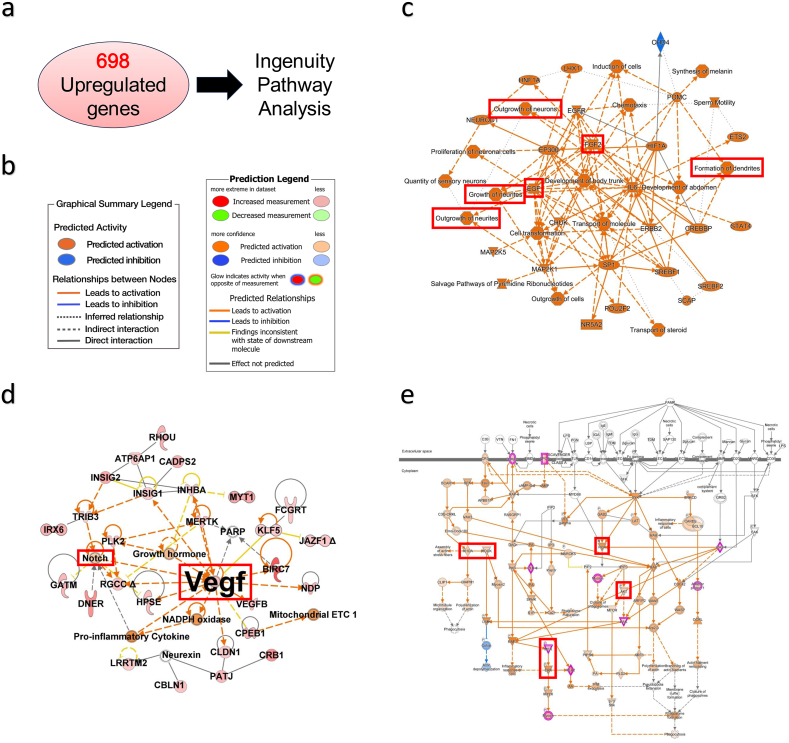
**Analysis of genes upregulated by trehalose. a** Ingenuity Pathway Analysis was performed using the 698 genes upregulated by 10 mg/ml trehalose treatment of 7 days. **b** Legends for the graphical summary and network analysis results. **c** Graphical summary of the effect of trehalose revealed by the upregulated genes. **d** Diagram showing the major network activated by trehalose. **e** Canonical pathways activated by trehalose. n = 4 for both control and trehalose groups.

To further investigate the effects of trehalose, we performed RNA-seq on hiPSC-NS/PCs cultured with or without trehalose (10 mg/ml) for 7 days. Trehalose induced the upregulation of 698 genes (FC > 1.2, p < 0.05). Using these sets of genes, we performed Ingenuity Pathway Analysis to elucidate the network and signaling pathways activated by trehalose (Fig. 2a and b). The graphical summary predicted that trehalose promotes cellular functions such as outgrowth of neurons, growth of neurites, and formation of dendrites through pathways activated by growth factors, such as fibroblast growth factor (FGF) and epithelial growth factor (EGF) (Fig. 2c). Among various growth factors, vascular endothelial growth factor (VEGF) was also shown to be playing a major role in the network activated by trehalose (Fig. 2d). In terms of canonical pathways, the Notch, RhoA/ROCK, PI3K/Akt, and MEK/ERK pathways were shown to be activated (Fig. 2d and e).

**Fig. 3:**
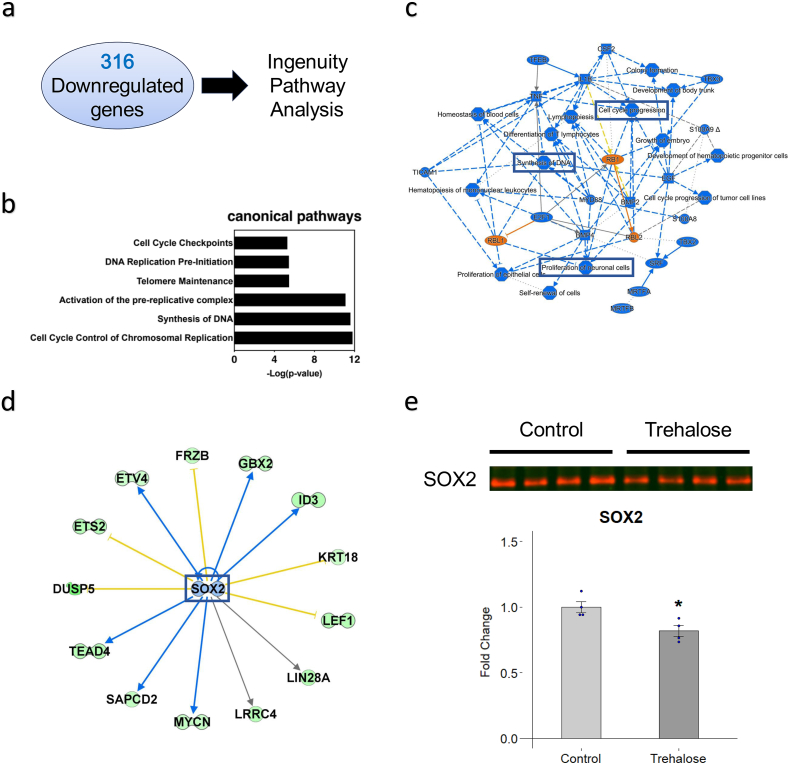
**Analysis of genes downregulated by trehalose. a** Ingenuity Pathway Analysis was performed using the 316 genes downregulated by 7 days of culture with trehalose (10 mg/ml). **b** Canonical pathways analysis of the downregulated genes. **c** Graphical summary of the Ingenuity Pathway Analysis using downregulated genes. **d** Decrease in Sox2 activity revealed by the Ingenuity Pathway Analysis. **e** Sox2 protein expression was assessed by western blotting. ∗p < 0.05 versus the control group according to Student-t-test. Data are presented as the mean ± SEM. n = 4 for both control and trehalose groups.

Conversely, 316 genes were downregulated in the presence of trehalose (FC < 0.8, p < 0.05). Our analysis revealed that trehalose treatment inhibits cell cycle progression, DNA synthesis, and proliferation of neuronal cells (Fig. 3a–c). Moreover, SOX2 activity was inhibited by trehalose (Fig. 3d). SOX2 is an important protein that regulates neural differentiation [19]. To evaluate the protein expression level of SOX2, Western blot analysis was performed using cells cultured with trehalose (10 mg/ml) for 7 days (Fig. 3e). The results revealed that trehalose treatment significantly decreased SOX2 protein expression (1.00 vs. 0.819, p = 0.023).

**Fig. 4:**
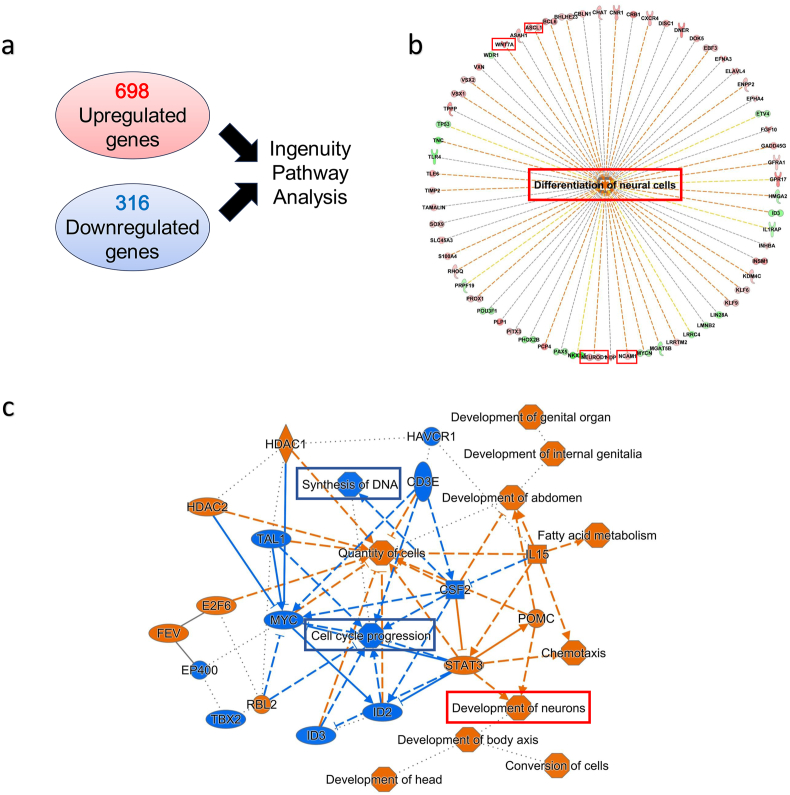
**Trehalose enhances neuronal differentiation and inhibits cell cycle progression. a** 698 upregulated genes and 316 downregulated genes detected by RNA-seq were integrated to perform Ingenuity Pathway Analysis. **b** Diagram showing the detected genes associated with the differentiation of neural cells. **c** Graphical summary of the Ingenuity Pathway Analysis. n = 4 for both control and trehalose groups.

To confirm these findings, we integrated both the upregulated and downregulated genes to analyze the cellular functions affected by trehalose (Fig. 4a). The expressions of multiple proneural genes such as *NEUROD1* and *ASCL1*, which encode transcription factors needed for the development of neurons [20], were shown to be elevated (Fig. 4b). In addition, multiple genes encoding important proteins for axonal branching and synaptogenesis, such as *WNT7A* and *NCAM1* [[21], [22], [23]], were elevated by trehalose-treatment. The graphical summary further demonstrated that trehalose promotes the development of neurons and differentiation of neural cells, while inhibiting cell cycle progression (Fig. 4c).

### 3.3 VEGFA expression is increased by trehalose treatment

**Fig. 5:**
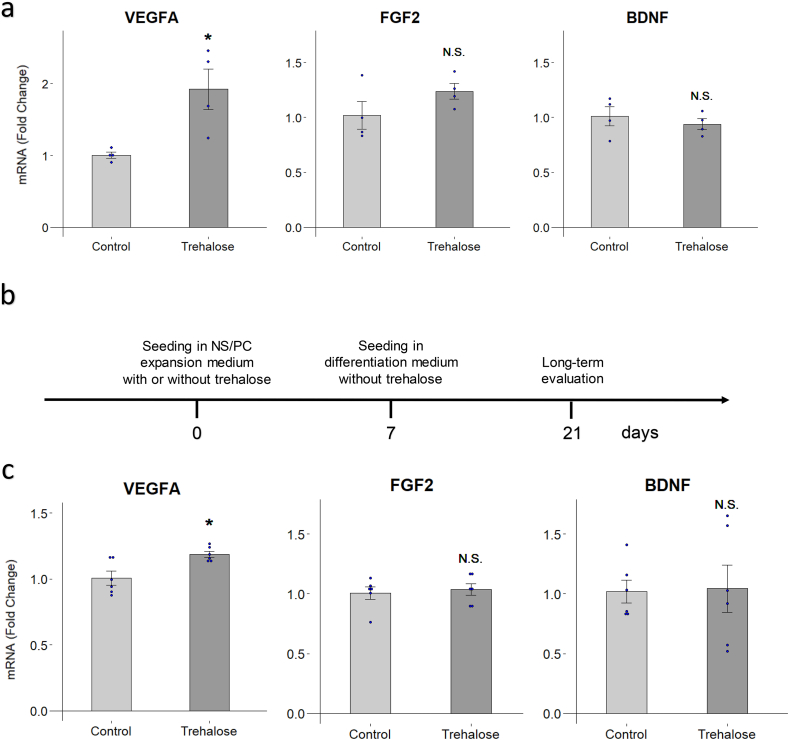
**Efficacy of trehalose on VEGFA, FGF2, BDNF expressions. a** VEGFA, FGF2, and BDNF expressions were assessed by qRT-PCR after hiPSC-NS/PCs were cultured with or without trehalose (10 mg/ml) for 7 days. n = 4 each. **b** Schematic for the evaluation of the long-term effects of trehalose on neural differentiation. **c** hiPSC-NS/PCs were differentiated for 14 days following the trehalose (10 mg/ml) treatment of 7 days. VEGFA, FGF2, and BDNF expressions were assessed by qRT-PCR. n = 6 each. ∗p < 0.05 versus the control group according to Student-t-test in **a** and **c**. Data are presented as the mean ± SEM.

To investigate the effect of trehalose on growth factors, we performed qRT-PCR on hiPSC-NS/PCs cultured with or without trehalose (10 mg/ml) for 7 days. From the results of RNA-seq (Fig. 2d), we focused on and measured the mRNA expressions of *VEGFA*, *FGF2*, and brain-derived neurotrophic factor (*BDNF*) (Fig. 5a). *VEGFA* is a member of the VEGF family, and is widely known as an angiogenic growth factor [24]. The cells treated by trehalose showed a significant increase in *VEGFA* expression (1.00 vs. 1.92, p = 0.045). Although *FGF2* and *BDNF* are both important neurotrophic factors in the central nervous system [25,26], there was no significant increase in their expression levels in the present study (*FGF2*: 1.02 vs. 1.24, p = 0.188; *BDNF*: 1.01 vs. 0.938, p = 0.486).

Next, to evaluate the long-term effect of trehalose on neural differentiation, we performed qRT-PCR on hiPSC-NS/PCs cultured *in vitro* in neural differentiation medium without trehalose for 14 days after the initial trehalose treatment of 7 days (Fig. 5b and c). The cells differentiated after trehalose treatment showed significant increase in *VEGFA* expression (1.01 vs. 1.19, p = 0.016), but there was no significant increase in *FGF2* and *BDNF* expression levels (*FGF2*: 1.01 vs. 1.04, p = 0.700; *BDNF*: 1.02 vs. 1.04, p = 0.917). These findings suggested that trehalose possesses the ability to enhance the expression of *VEGFA* even after neuronal differentiation.

### 3.4 Trehalose enhances neurite outgrowth

**Fig. 6:**
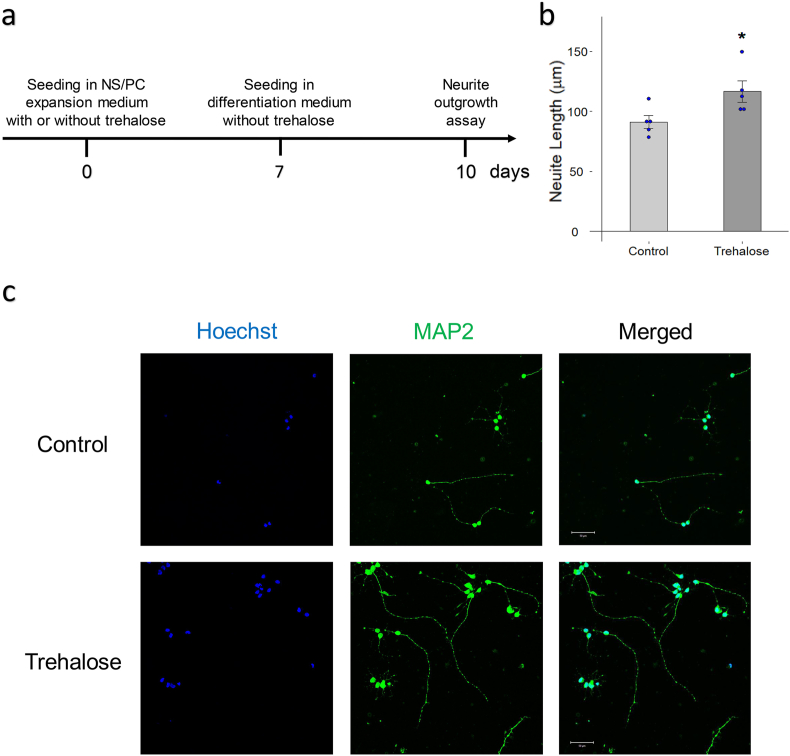
**Trehalose enhances neurite elongation. a** Schematic for the neurite outgrowth assay. **b** Quantitative neurite length analysis of hiPSC-NS/PCs that were differentiated for 3 days following 7 days of trehalose (10 mg/ml) treatment. n = 5 each. ∗p < 0.05 versus the control group according to Student-t-test. Data are presented as the mean ± SEM. **c** Representative image of immunostaining for MAP2 with Hoechst from the control group (upper) and trehalose group (lower) at 3 days after seeding in differentiation medium. Scale bar = 50 μm.

To further explore the effects of trehalose on neuronal differentiation, we performed a neurite outgrowth assay on cells differentiated without trehalose for 3 days following 7 days of trehalose treatment (Fig. 6a–c and Table S1). MAP2 is a protein required for dendrite elongation and is a widely known marker for neurons [27]. The results showed that MAP2-positive cells treated by trehalose showed significant increase in neurite length (91.1 μm vs. 116.5 μm, p = 0.039).

## 4 Discussion

In this study, we investigated the effects of trehalose on cultured hiPSC-NS/PCs. Our results showed that trehalose treatment upregulated *CDKN1A* expression and decreased cell viability, suggesting the induction of cell cycle arrest. Western blotting and differentiation assays demonstrated the downregulation of SOX2 protein expression and enhancement of neurite outgrowth, indicating that trehalose can augment the differentiation of hiPSC-NS/PCs. These results were consistent with the findings from analyzing the genes revealed by RNA-seq. Moreover, trehalose-treated cells showed a long-term increase in *VEGFA* expression, even after the depletion of trehalose in the culture medium. Thus, this is the first study to show that trehalose can amplify the neuronal differentiation and secretion of *VEGFA* in hiPSC-NS/PCs.

Martano et al. reported that trehalose enhanced arborization in neurons, which is compatible with our results obtained from hiPSC-NS/PCs. However, the mechanism underlying the effect of trehalose on neuronal differentiation remains elusive. In our study, we observed that trehalose treatment enhanced *CDKN1A* expression which is known for its function in cell cycle arrest. However, several reports have shown the involvement of p21, a protein encoded by the *CDKN1A* gene, not only in cell cycle regulation, but also in neural differentiation [[28], [29], [30]]. Tanaka et al. reported that cytoplasmic expression of p21 promoted neurite outgrowth [29. Moreover, p21 negatively regulates the expression of SOX2 in neural stem cells by directly binding to the enhancer of *Sox2* gene [30]. In fact, our results showed that SOX2 protein expression is decreased (Fig. 3d). Because SOX2 plays a role in sustaining the undifferentiated state of NS/PCs, we hypothesize that p21 and SOX2 are involved in the influence trehalose has on neuronal differentiation.

There have been several studies that show the direct involvement of *VEGFA* in neurogenesis [31,32]. Rosenstein et al. reported that VEGF mediates neurite growth and maturation by activating the MEK/ERK and PI3–K/Akt signaling pathways through VEGFR2/Flk-1 receptors [33]. Our results revealed that trehalose treatment of hiPSC-NS/PCs can promote a long-term increase in *VEGFA* expression during differentiation. Moreover, canonical pathway analysis from the RNA-seq suggested that trehalose activates both MEK/ERK and PI3–K/Akt signaling pathways (Fig. 2e). Therefore, we speculate that trehalose promotes neurite elongation and neuronal differentiation by enhancing the expression of *VEGFA*.

Cell transplantation therapy is extensively studied as a promising approach for treating neurological diseases, given the limited regenerative capacity of the central nervous system. For instance, numerous studies using animal models have demonstrated the potential of hiPSC-NS/PC transplantation for spinal cord injury (SCI). Among the mechanisms of its therapeutic efficacy, neuronal differentiation of transplanted hiPSC-NS/PCs in the SCI region is particularly an important factor in enhancing synaptic formation between the grafted cells and host tissue [[34], [35], [36], [37]]. Furthermore, we previously reported that transplanted hiPSC-NS/PCs secrete VEGF, which is crucial for inducing angiogenesis and promoting functional recovery [38]. Similarly, the importance of neurons and VEGF deriving from the transplanted neural stem cells has been reported in other nervous system diseases such as amyotrophic lateral sclerosis (ALS) and stroke [[39], [40], [41]]. In the present study, we demonstrated that treatment of hiPSC-NS/PCs with trehalose enhances neurite outgrowth and promotes long-lasting *VEGFA* expression. We therefore speculate that trehalose treatment has the potential to improve various types of stem cell therapies used for neurological diseases.

Our results showed that trehalose at both 10 and 40 mg/ml upregulates the expression of *CDKN1A*, but cell viability was significantly reduced only at 40 mg/ml. Because *CDKN1A* is known to cause cell cycle arrest [18], trehalose could inhibit the proliferation rate, which may cause a decrease in cell viability after 7 days of culture. However, the *CDKN1A* expression at 40 mg/ml tended to be lower than at 10 mg/ml, which indicates that the decrease in cell viability was caused not only by *CDKN1A* expression but also by a high concentration of trehalose that induced cell death in hiPSC-NS/PCs. Similar to our results, a previous study reported that trehalose showed significant toxicity at 250 mM (85.5 mg/ml) during the culture of iPSC-derived neurons [42]. In contrast, Muto et al. reported that trehalose treatment of fibroblasts induces the upregulation of growth factors, such as *VEGFA* and *FGF2*, through the *CDKN1A*/p21 pathway in a dose-dependent manner at an optimal concentration of 100 mg/ml [11]. Together, these reports suggest that hiPSC-NS/PCs are relatively vulnerable to the cytotoxicity of trehalose. Hence, it is necessary to carefully consider the concentration of trehalose when applying to other cell types.

Several studies have shown that oral administration of trehalose can have therapeutic benefits through the induction of autophagy in multiple diseases such as myocardial infarction, ALS, SCI, and Parkinson's disease [[43], [44], [45], [46]]. Furthermore, intrathecal injection of trehalose promotes functional recovery in rat SCI models through the suppression of oxidative stress and inflammation [47,48]. These reports indicate that, in combination with the cell transplantation therapy, post-transplantation administration of trehalose may represent a viable adjunctive treatment strategy. However, our results showed that trehalose treatment enhanced neuronal differentiation and VEGF secretion even after withdrawal of trehalose from the culture medium, suggesting that the use of trehalose before cell transplantation could improve cell transplantation therapy as well. We believe that the use of trehalose pre-transplantation is ideal because it ensures that the transplanted cells are treated with an optimal concentration of trehalose. To clarify the effect of post-transplantation trehalose treatment on neuronal differentiation and VEGF secretion, further research is required to examine the methods of delivering trehalose *in vivo*.

### 4.1 Limitations

The present study has several limitations. Our results showed that trehalose can promote neuronal differentiation of hiPSC-NS/PCs *in vitro*, as shown by transcriptome analysis and morphological analysis of the neurites. However, we could not clarify the clinical applicability of trehalose in cell transplantation therapy *in vivo*. In addition, we could not elucidate the effect of trehalose on other lineages deriving from neural stem cells such as astrocytes and oligodendrocytes, because the hiPSC-NS/PCs used in this study are highly neurogenic (Fig. S1). Moreover, the effect of cell cycle arrest of hiPSC-NS/PCs on cell transplantation remains unknown and further research is required.

## 5 Conclusion

We showed that low concentrations of trehalose can enhance p21 and *VEGFA* expression, promoting neuronal differentiation in hiPSC-NS/PCs. We believe that these properties of trehalose could contribute to the future advancement of stem cell transplantation therapy.

## Declaration of competing interest

The authors declare that they have no known competing financial interests or personal relationships that could have appeared to influence the work reported in this paper.
