# Supplementary material for: Trehalose enhances neuronal differentiation with VEGF secretion in human iPSC-derived neural stem/progenitor cells
Source: Regen Ther. 2025 Jun 26;30:268–77. doi: 10.1016/j.reth.2025.06.012 (PMC12246652; doi:10.1016/j.reth.2025.06.012)
Supplement: Multimedia component 1 [file mmc1.pptx]

## Slide 1
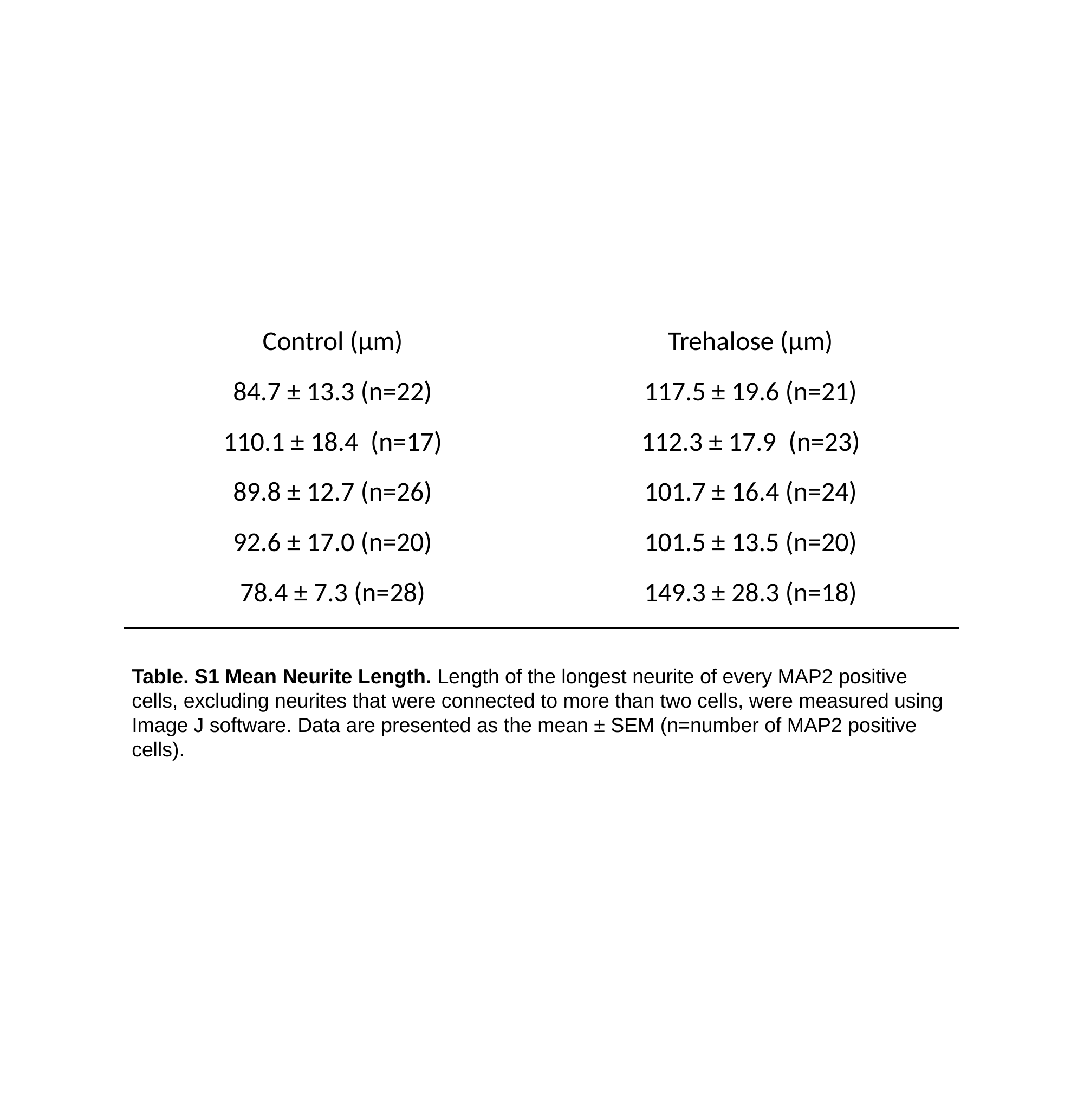

| Control (μm) | Trehalose (μm) |
| --- | --- |
| 84.7 ± 13.3 (n=22) | 117.5 ± 19.6 (n=21) |
| 110.1 ± 18.4 (n=17) | 112.3 ± 17.9 (n=23) |
| 89.8 ± 12.7 (n=26) | 101.7 ± 16.4 (n=24) |
| 92.6 ± 17.0 (n=20) | 101.5 ± 13.5 (n=20) |
| 78.4 ± 7.3 (n=28) | 149.3 ± 28.3 (n=18) |
Table. S1 Mean Neurite Length. Length of the longest neurite of every MAP2 positive cells, excluding neurites that were connected to more than two cells, were measured using Image J software. Data are presented as the mean ± SEM (n=number of MAP2 positive cells).

## Slide 2
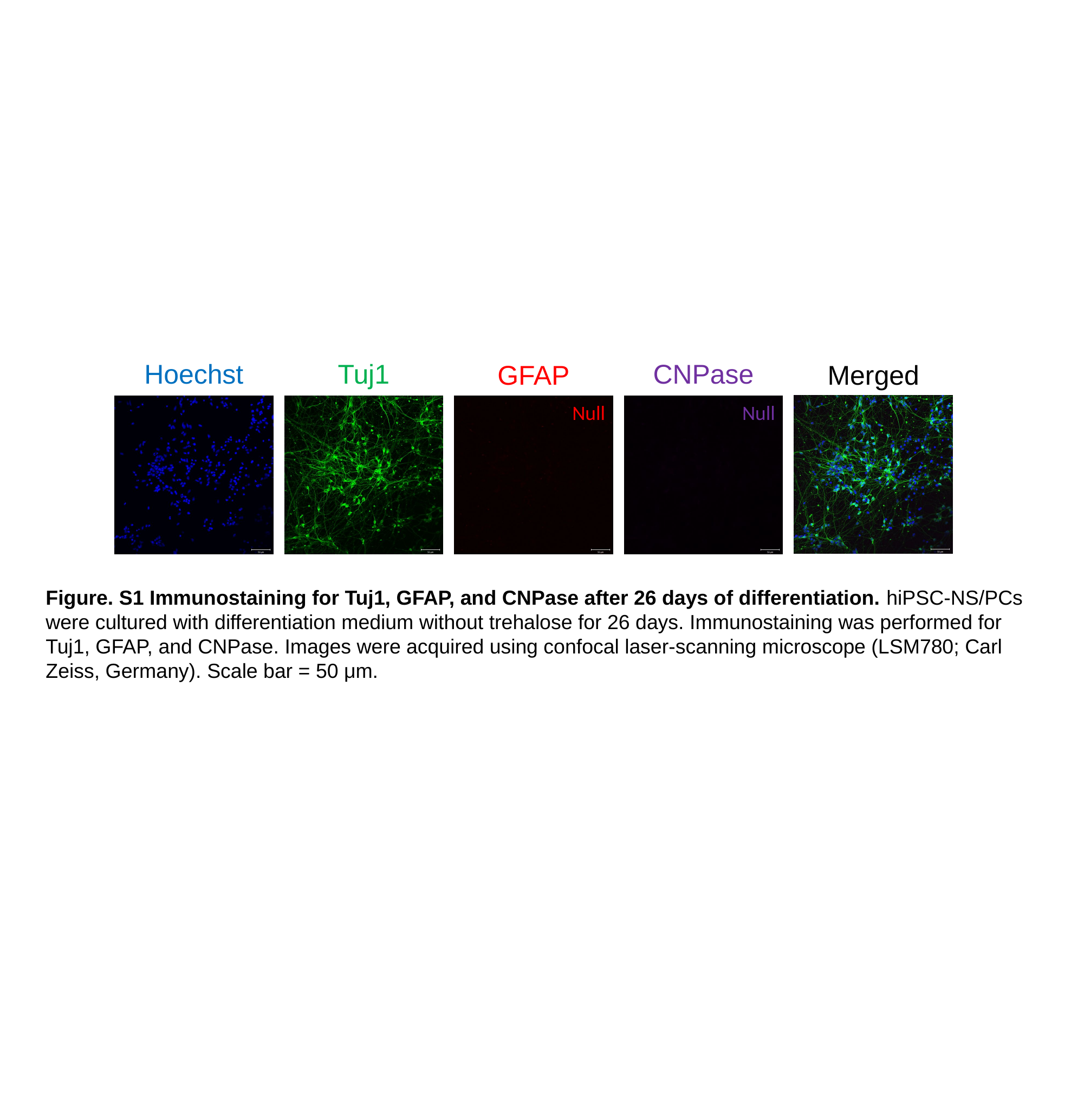

Hoechst
Tuj1
CNPase
GFAP
Merged
Null
Null
Figure. S1 Immunostaining for Tuj1, GFAP, and CNPase after 26 days of differentiation. hiPSC-NS/PCs were cultured with differentiation medium without trehalose for 26 days. Immunostaining was performed for Tuj1, GFAP, and CNPase. Images were acquired using confocal laser-scanning microscope (LSM780; Carl Zeiss, Germany). Scale bar = 50 μm.
